# Translation of preclinical ethnomedicine data in LMICs: the example of rooibos

**DOI:** 10.3389/fphar.2023.1328828

**Published:** 2023-12-19

**Authors:** Lesha Pretorius, Carine Smith

**Affiliations:** Experimental Medicine, Department of Medicine, Faculty of Medicine and Health Sciences, Stellenbosch University, Cape Town, South Africa

**Keywords:** rooibos, clinical trial, human, challenges, regulatory systems, gut-brain, inflammation

## Abstract

All disease, but especially non-communicable diseases, are related to dysfunction of one or more regulatory systems. In developing countries, long-term management of patients with chronic diseases has many challenges and is generally not financially viable, but Africa in particular, which is rich in diverse ethnomedicines presents a more feasible long-term therapeutic approach in this niche. However, despite comprehensive preclinical investigations on numerous plant-derived candidate medicines, only a small portion of these reach the patient as recognised medicines. In this review, we use the example of rooibos (*Aspalathus linearis* (Burm.f.) R. Dahlgren)–which is globally consumed as aromatic, caffeine-free tea–to illustrate the hurdles that need to be overcome in the low-to middle-income countries, before progression of ethnomedicines to official treatment regimens can be achieved. In terms of methodology, regulatory system focused rooibos papers indexed on PubMed for the past three decades (n = 112) were accessed. Papers reporting duplication of previous results were excluded, as well as review papers. Topics covered includes the high standard of ethnomedicine drug discovery and efficacy testing research performed in Africa (and South Africa in particular in the case of rooibos), the potential bias in terms of preclinical research focus, ethnomedicine ownership and the requirement for independent clinical trial coordination and/or management.

## Introduction

A recent fact sheet by the World Health Organisation reported 37% of deaths in Africa to result from non-communicable diseases, with 63% of deaths classified as premature (i.e., before the age of 70) (Wangou et al., 2022). Even more alarmingly in terms of burden on public health systems, is that the International Diabetes Federation reported a diabetes incidence in Africa of 1 in 22 (IDF, 2021). Furthermore, in countries with increased rates of urbanisation and sedentary lifestyles, such as South Africa, incidence may be as high as 1 in 10 (Erzse et al., 2019). In 2015, the National Institute of Health estimated the cost of diabetes alone in Sub-Saharan Africa at USD 19.45 billion annually–1.2% of gross domestic product (Erzse et al., 2019). From these statistics, it is clear that long-term management of chronic patients using mainstream pharmaceuticals is neither financially viable, nor entirely successful.

Africa is rich in candidate medicines that were initially identified largely via indigenous knowledge systems (Sprent et al., 2010). Thus, Africa should be strategically poised at the frontier of medicine development to address the plight of the chronically ill in Africa and other low-to middle-income countries (LMICs) globally. However, despite African ethnomedicine riches, only a fraction of *worthy* candidate medicines reaches the point where they are officially recognised and registered as mainstream medicines, which would allow their incorporation into policy documents and widespread application in public sector medical centres.

With this mini-review, using the example of Rooibos (a globally consumed tea, prepared from *Aspalathus linearis* (Burm.f.) R. Dahlgren), we unpack some of the strengths and weaknesses of ethnomedicine research practices such as the research group bias and the relative lack of clinical trials. We also make suggestions on how the more unique challenges faced by African drug discovery and medicines development scientists, on their road to recognition of ethnomedicines as equivalent treatment regimes in disease, may be addressed. These include safety concerns, ethnomedicine ownership, stagnation of medicines development at the preclinical phase.

## Rooibos: doomed to fail or on the road to success?

Rooibos is a herbal plant indigenous to South Africa and is globally consumed as tea, for its aromatic taste and health benefits. Rooibos is commercially available in two forms, unfermented (green) and fermented. Although the unfermented rooibos generally exhibits higher potency–likely due to its higher polyphenol content–similar health promoting attributes have been reported for both. Thus, distinction between the two types was not relevant for the purpose of this review.

Rooibos was chosen as example ethnomedicine, as it has been assessed in the context of modulation of many regulatory systems, which are central to the physiological dysregulation resulting in chronic disease. Furthermore, a robust body of literature generated by many different research groups exist on its potential as medicine in both a preventative and therapeutic approaches across various models and diseases, and it has been thoroughly characterised in terms of active constituents.

### Evidence of general antioxidant and anti-inflammatory efficacy

Rooibos is widely known and consumed for its antioxidant activity. The antioxidant mechanism of rooibos seems multi-pronged: firstly, it has demonstrated ability to scavenge free radicals in a manner similar to vitamin C and E (Snijman et al., 2009; Lopez et al., 2022), a mechanism ascribed to mainly two active ingredients–aspalathin and nothofagin (Bramati et al., 2003; Joubert et al., 2005; Chen et al., 2013; Canda et al., 2014). In fact, rooibos has such high potency that pro-oxidant outcome have been reported for extracted (i.e., concentrated) rooibos when administered in the absence of disease or conditions of oxidative stress (Joubert et al., 2005). Secondly, rooibos constituents quercetin, aspalathin and catechin inhibit lipid peroxidation (Snijman et al., 2009), a mechanism likely responsible for its reported membrane-protective effects of rooibos (Ulicna et al., 2019; Pretorius and Smith, 2022). A multitude of studies in cell-free systems and in cell culture have also demonstrated improved antioxidant enzyme activities (particularly for catalase and superoxide dismutase (SOD)) (Waisundara and Hoon, 2015) and mitochondrial bioenergetics (Mthembu et al., 2021).

Redox benefits of rooibos administration have also been successfully translated into various *in vivo* settings. In rodent models, these benefits included increased rodent serum SOD and decreased urinary 8-Oxo-dG levels (Baba et al., 2009), attenuation of increases in plasma and hepatic MDA and decreases in whole blood and liver GSH:GSSH ratios (Ajuwon et al., 2014; van der Merwe et al., 2015), restoration of t-BHP- and CCl4-induced reduction of liver antioxidant status (Canda et al., 2014; Ulicna et al., 2019) and improved mitochondrial bioenergetics (elevated mitochondrial enzymes, improved capacity of the electron transport chain and increased energy production) (Ulicna et al., 2019). Also in invertebrate models such as *C. elegans*, rooibos supplementation decreased juglone-induced acute oxidative damage and extended the lifespan of *Caenorhabditis elegans* in a high glucose environment (Chen et al., 2013). Using quantitative real-time PCR, the authors demonstrated that aspalathin targeted stress and aging related genes as a potential mechanism of action.

The antioxidant capacity of rooibos also links closely to an anti-inflammatory outcome. In this context, data from *in vitro* models of inflammation associate rooibos administration with improved tight junction protein expression and interaction status to maintain endothelial cellular structure and barrier integrity (Ku et al., 2015; Lee and Bae, 2015; Pretorius and Smith, 2022), modulation of PGE2 synthesis (Hedbrant et al., 2022), inhibition of basophil activation (Pedretti and Peter, 2020) and an inhibitory effect on concentrations of pro-inflammatory mediators–such as IL-1α, IL-6, IL-8, VCAM-1 and ATF4 gene expression (Lawal et al., 2019b), as well as IFN-γ, IL-12, IL-2, IL-17a, TNF-α, IL-8, IL-6, IL-1β and CXCL10 (Nehme et al., 2023)–and increased anti-inflammatory cytokine, IL-10 (Ichiyama et al., 2007). In addition, the flavonoid actives aspalathin and nothofagin were reported to prevent the expression of cell adhesion molecules and transendothelial migration of neutrophils in LPS- and high glucose-exposed cells (Ku et al., 2015; Lee and Bae, 2015), while other rooibos constituents quercetin, luteolin and chrysoeriol reportedly inhibited antigen- and calcium ionophore-stimulated degranulation in basophils (Morishita et al., 2019).

In the inflammation context, data from *in vivo* studies again corroborates *in vitro* data. Here, rooibos-treated rodents exposed to LPS exhibited blunted TNF-α and IL-6 responses (Ajuwon et al., 2014), while rooibos pre-treatment reversed DEP-induced increases in IL-8, TNF-α, NF-κB and IL-1β and decreases in IL-10 gene expression (Lawal et al., 2019a). Also in response to the hepatotoxic challenge CCl4, a reduced NF-κB expression was reported in rooibos-administered rats (Gabuza et al., 2022). In terms of specific flavonoids, mice treated with aspalathin and nothofagin showed blunted inflammatory responses (TNF-α, IL-6, NF-κB activation, endothelial hyperpermeability and leukocyte migration) to both LPS- and high glucose challenge (Ku et al., 2015; Lee and Bae, 2015).

In addition to general demonstrations of antioxidant and anti-inflammatory actions of rooibos, potential for therapeutic effect in specific disease states have also been reported. A large number of rodent studies have linked antioxidant and anti-inflammatory effects of rooibos to cardiovascular health in particular, including benefits in terms of both vasculature and myocardial tissue (Katengua-Thamahane et al., 2014; Ku et al., 2015; Lee and Bae, 2015; Obasa et al., 2020; Smit-van Schalkwyk et al., 2020; Smit et al., 2022).

Despite the solid pre-clinical database, we could find only one clinical trial that focused on elucidating benefits of rooibos supplementation in a vulnerable population - in subjects at risk for coronary heart disease, rooibos consumption improved plasma lipid profiles (decreased LDL-cholesterol and triacylglycerols; increased HDL-cholesterol) and redox status (decreases in lipid peroxidation (conjugated dienes); increases in glutathione and GSH:GSSH ratio), suggesting a beneficial effect of rooibos to reduce risk factors implicated in developing cardiovascular disease (Marnewick et al., 2010).

### Endocrine (metabolic) efficacy

Given the high incidence of diabetes in developing countries, it is no surprise that a robust body of rooibos research exist in this context. One of the first reports on rooibos illustrated it to ameliorate diet-induced metabolic disturbances (including lipid profile, adipocyte size and hepatic steatosis) in hyperlipidemic mice (Beltran-Debon et al., 2011), suggesting rooibos to have a role as preventative modality in metabolic disease. Aspalathin was named one of the most important active ingredients in rooibos, facilitating a beneficial outcome in terms of glucose levels and insulin sensitivity in *db/db* mice (Kawano et al., 2009).

These positive reports sparked research focused on assessing the anti-diabetic potential of rooibos, which were largely led by researchers in South Africa, where rooibos grows naturally. Research expanded on the first studies, illustrating rooibos to enhance glucose uptake into (C2C12) murine skeletal muscle myotubules and to lower blood glucose with a potency similar to that of metformin (Muller et al., 2012) in streptozotocin (STZ)-induced diabetic rats, with the constituent polyphenols aspalathin and rutin demonstrated to act in synergy to achieve these effects. In addition, the constituent phenylpyruvic acid-2-O-glycoside (PPAG) was illustrated to play a major role in improving glucose metabolism in Chang (human hepatic) cells (Muller et al., 2013). Follow-up studies by the same group expanded on the antidiabetic potential of rooibos by demonstrating its ability to prevent experimentally induced insulin resistance and glucose-associated detrimental effects in several different cell types, including C2C12 murine skeletal muscle myotubules (Mazibuko et al., 2013), H9c2 murine embryonic cardiomyocytes (Dludla et al., 2016), 3T3-L1 murine adipocytes (Mazibuko et al., 2015) and C3A human liver carcinoma cells (Mazibuko-Mbeje et al., 2019) via antioxidant-dependent and independent mechanisms (Mazibuko-Mbeje et al., 2019; Mazibuko-Mbeje et al., 2022). In addition, rooibos was shown to reduce lipid accumulation in 3T3-L1 liver cells (Sanderson et al., 2014), suggesting a further beneficial anti-diabetic mechanism, while aspalathin specifically was demonstrated to protect pancreatic β-cells from *in vitro* glucotoxicity (Moens et al., 2020) and lipotoxicity (Moens et al., 2022).

The robustness of the comprehensive preclinical data set generated by this group, is evident from the similarly positive data by other research groups illustrating benefits of rooibos in other rodent models of diabetes (Son et al., 2013; Kamakura et al., 2015), as well as in L6 myoblasts and pancreatic β-cells (Kamakura et al., 2015; Himpe et al., 2016). New technology was recently used to confirm known effects and mechanisms, e.g., via targeted cellomics screening (Pringle et al., 2021) and network pharmacology- and molecular dynamics simulation-based bioprospecting (Akoonjee et al., 2022). However, despite the robust preclinical data, translation into human models are still lacking.

Rooibos and its constituents have not yet been investigated for other potential endocrine effects to the same extent. Only one group has reported on the effect of rooibos and some constituents on adrenal steroidogenesis. Indeed, an inhibitory effect of rooibos on specific P450 enzymes was demonstrated in fibroblast-like primate kidney (COS-1) cells, as well as a general downregulation of synthesis of corticosteroid and aldosterone precursors in H295r human adrenocarcinoma cells (Schloms et al., 2012; Schloms and Swart, 2014). This data aligns with the “calming” effect anecdotally ascribed to rooibos. Encouragingly, this group also reported on parallel supplementation studies in humans (the same cohort study reported on in Marnewick et al. (2010) and rats. These *in vivo* studies jointly provided evidence of decreased glucocorticoid synthesis after rooibos consumption–most notably a lower corticosterone:testosterone ratio in rodents, and a lower cortisol:cortisone in humans (Schloms and Swart, 2014).

### Neglected regulatory systems

Although rooibos is anecdotally acclaimed for its calming effect few studies have investigated the effect of rooibos on the brain and nervous system and associated behavioural outcomes. Somewhat disappointingly, most *in vitro* studies in this context–albeit with positive results–seem largely focused on antioxidant effects (Hong et al., 2014; Fisher et al., 2020; Ma et al., 2020; Lopez et al., 2022; Brasil et al., 2023). Only two of these expanded their investigation and also reported different modulatory mechanisms in the context of serotonin metabolism (Hong et al., 2014; Lopez et al., 2022), which provides some additional mechanistic support for an anxiolytic or calming effect.

However, the limited scope of these studies may be a limitation of neuronal cell models, as *in vivo* studies in rodents reported a larger variety of beneficial functional outcomes for rooibos, such as improved long-term spatial memory that was associated with increased striatal dopamine and 3-methoxytyramine (dopamine metabolite) levels, but not excessive motor activity (Pyrzanowska et al., 2019), as well as reduced anxiety and increased exploratory behaviour (Pyrzanowska et al., 2021). These findings have also been corroborated in zebrafish larvae (Lopez et al., 2022), in which a significant GABA agonist effect of rooibos was elucidated.

Collectively, available literature shows neuroprotective potential mediated by an increase in antioxidant enzyme activity (Akinrinmade et al., 2017) and/or modulation of neurotransmission (Lopez et al., 2022), which translates to improved behavioural outcome (Pyrzanowska et al., 2019; Pyrzanowska et al., 2021; Lopez et al., 2022). However, in this context, literature is still relatively sparse.

Somewhat related to this, when considering the known signalling between the brain and the gut, the tradition route of rooibos consumption (orally as a tea), as well as the recognition of the microbiome as additional regulatory system, it seems logical to also consider effects of rooibos in the gut. Indeed, in the rooibos context, emerging evidence suggests a prebiotic-like modulation of the gut microbiome. In vervet monkeys, rooibos normalised Firmicutes to Bacteroidetes ratio to rescue high-fat high-sugar diet-induced metabolic dysregulation (Mangwana, 2020), and increased relative abundance of beneficial bacterial species (*Faecalibacterium prausnitzii, Prevotella stercorea* and *P. copri, Akkermansia muciniphia, Bacteroides intestinalis, Desulfovibrio piger* and *Bifidobacterium adolescentis*). More recent *in vitro* studies confirmed increased probiotic microbial growth and favourably modulated secretome trace amine content to promote gut health (Pretorius et al., 2022; Pretorius and Smith, 2022). Taken together, changes in the gut microbiome are likely to influence host regulation and homeostasis and as such, the potential benefit conferred by rooibos supplementation necessitates additional robust investigation.

## Requirements for transformation from tea to treatment

**FIGURE 1:**
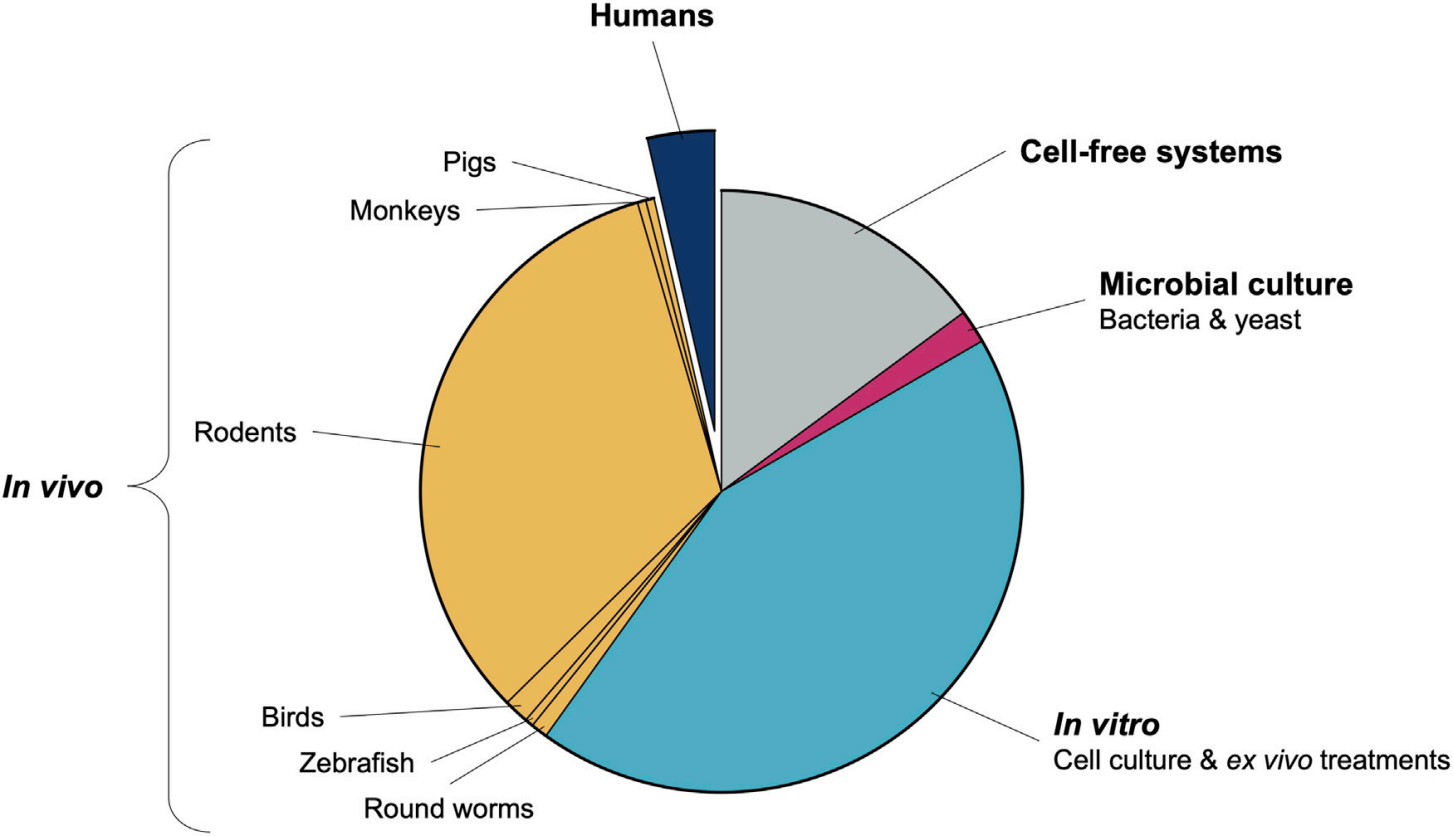
Relative popularity of models used in published rooibos-related health research studies.

A few facts are clear from the rooibos context, which may be extrapolated more broadly to ethnomedicine in Africa. Firstly, existing data on rooibos is largely positive and reproducible across various experimental models and research groups, suggesting that rooibos has significant merit as candidate ethnomedicine. Secondly, perhaps due to limited scope of interest or specific expertise of isolated research groups, data on specific systems (such as redox) are almost excessive, while relatively lacking for others. Thirdly, despite almost two decades of preclinical research, translation into human trials is near absent (Figure 1), suggesting absence of a coordinated, systematic approach to medicine development. Moreover, the human studies that have been performed are often limited (few parameters assessed on available samples) and self-serving to the research group interest. We provide some suggestions on how these issues may be addressed going forward.

### Addressing safety concerns

A potential obstacle to human testing of ethnomedicines in general, is concerns around safety. As would be the case for any mainstream candidate pharmaceutical, specific pharmacological and pharmacovigilance data on toxicity, risk for drug-interaction, ADME profile, risk of off-target adverse events, etc. would be required for any ethnomedicine to be registered as a mainstream pharmaceutical. Unfortunately, it seems as if this approach is deemed financially unfeasible in the African ethnomedicine environment, where marketing and exporting plant-derived products as food supplements are already generating substantial revenue.

In the case of rooibos, significant safety data has been generated mostly by preclinical researchers using *in vitro* models to address this. In terms of cytochrome p450 (CYP450) and other enzymes related to enzymes related to drug metabolism, data consistently suggest that rooibos may affect drug bioavailability (Marnewick et al., 2003; Matsuda et al., 2007; Fantoukh et al., 2019; Patel et al., 2019). This data has been confirmed in rodents in the context of hypoglycemic and hypolipidemic treatments (including metformin and atorvastatin) (Patel et al., 2019), of which atorvastatin is metabolised by CYP450 enzymes. Importantly, no herb-drug interaction was reported for metformin and rooibos co-treatment (Patel et al., 2019), suggesting this may be an avenue in to pursue in humans (Dludla et al., 2018). All *in vivo* studies seem to indicate potentiation of drug action by rooibos, especially that of atorvastatin, i.e., lower doses of pharmaceuticals may be required for therapeutic effect when used in combination with rooibos. A compounding approach may therefore limit incidence of adverse drug effects, as well as lowering treatment costs, illustrating both the importance of safety testing and the significant role that ethnomedicines could play in patient management, especially in poor countries. However, pre-clinical researchers rarely have the means or intent of progressing to clinical trials, which halts the transition to clinical trials at this point.

### Progression to clinical trials

**FIGURE 2:**
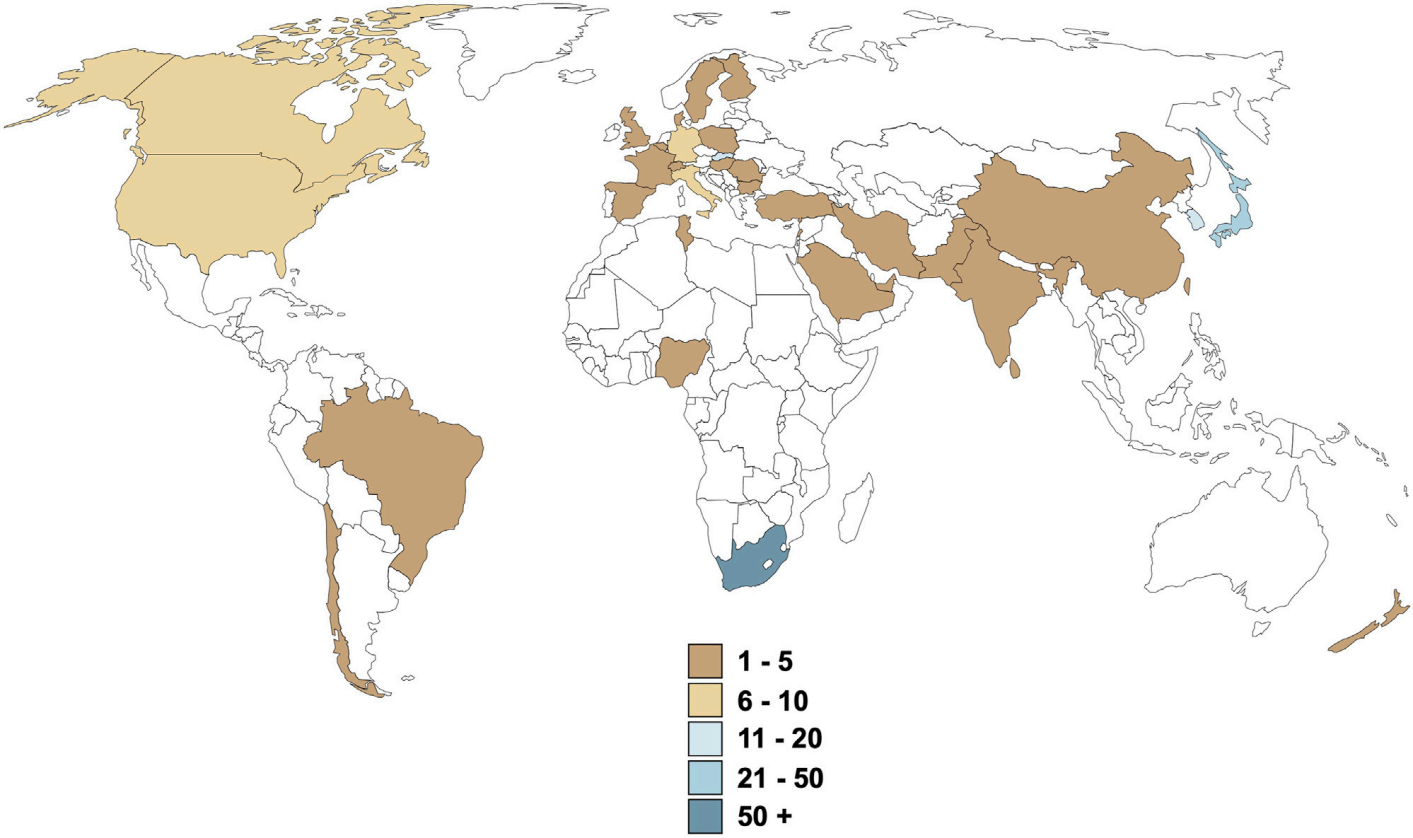
Origin of health-related rooibos research over the last 3 decades. Values are representative of the number of publications produced, excluding literature reviews and case reports.

Although reproducibility of research data should be confirmed, the decision that pre-clinical data is sufficient to warrant clinical trials in humans, should be made timeously to prevent stagnation of medicines development at the preclinical stage. We believe that the cost, in terms of human resources, monetary expense, legislative compliance and administrative resources, is only one reason for the relative lack of throughput. In the case of rooibos, clear research “silos” are evident, although there is clearly global interest in rooibos (Figure 2), suggesting lack of a coordinated effort. Given the fact that identification of commercial ownership of an ethnomedicine is a complex negotiation, especially when no refinement or extraction process is required for therapeutic potency, the manufacturing pharmaceutical industry is understandably not interested in coordinating or funding research on any particular ethnomedicine. Although indigenous ownership has been acknowledged for rooibos via traditional knowledge and access-benefit sharing regulations leading to the Rooibos Benefit Sharing Agreement (UEBT, 2017; Schroeder et al., 2020; Meyer and Naicker, 2023), this resulted from a tedious 6-year administrative process. While the signing of this agreement has positively impacted the future of rooibos as potential medicine, this route is not the norm for the majority of ethnomedicines, where bioprospecting is neglected and ethnomedicines are sold as dietary supplements only. There thus exists a dire need for a knowledgeable and suitably equipped entity to take charge of the ethnomedicine pipeline from bench to bedside (as is currently attempted for rooibos by the South African Rooibos Council).

The World Health Organization (WHO) seems to be taking the lead in recognition of traditional medicines, with the new WHO Global Centre for Traditional Medicine scheduled to open its doors in 2024 (WHO, 2023). While the priority of this centre is not yet clear, the expectation and hope is that this centre may play a role in “talent identification” of ethnomedicines, connecting role players with various expertise and backgrounds to construct multidisciplinary, equipped teams, as well as facilitating the progression of candidate medicines through a clinical trial process.

## Conclusion

Clearly, preclinical researchers in Africa are doing high quality research to aid in identification of potential ethnomedicines and to provide scientific support for traditional practices which may contribute to mainstream medical care. However, given the dire state of long-term chronic patient management in poor countries, we believe that a centralised agency is required to improve the coherence of currently disjointed efforts, in order to achieve affordable medicine and improvement of quality of life for all.
